# Impaired keratinization activity in perianal Crohn’s fistulas is associated with poor prognosis

**DOI:** 10.1093/ecco-jcc/jjag092

**Published:** 2026-06-30

**Authors:** Marte A J Becker, Pim J Koelink, Sarah Ouahoud, Sander M Meisner, Vanesa Muncan, Gerald Nabozny, Frank Li, Job Saris, Willem A Bemelman, Geert R D’Haens, Christianne J Buskens, Manon E Wildenberg

**Affiliations:** Department of Gastroenterology and Hepatology, Amsterdam Gastroenterology Endocrinology and Metabolism, Amsterdam UMC, University of Amsterdam, Amsterdam, The Netherlands; Tytgat Institute for Liver and Intestinal Research, Amsterdam Gastroenterology and Metabolism, Amsterdam UMC, University of Amsterdam, Amsterdam, The Netherlands; Tytgat Institute for Liver and Intestinal Research, Amsterdam Gastroenterology and Metabolism, Amsterdam UMC, University of Amsterdam, Amsterdam, The Netherlands; Tytgat Institute for Liver and Intestinal Research, Amsterdam Gastroenterology and Metabolism, Amsterdam UMC, University of Amsterdam, Amsterdam, The Netherlands; Tytgat Institute for Liver and Intestinal Research, Amsterdam Gastroenterology and Metabolism, Amsterdam UMC, University of Amsterdam, Amsterdam, The Netherlands; Department of Gastroenterology and Hepatology, Amsterdam Gastroenterology Endocrinology and Metabolism, Amsterdam UMC, University of Amsterdam, Amsterdam, The Netherlands; Tytgat Institute for Liver and Intestinal Research, Amsterdam Gastroenterology and Metabolism, Amsterdam UMC, University of Amsterdam, Amsterdam, The Netherlands; Department of Immunology and Respiratory Diseases Research, Research and Development Boehringer-Ingelheim, Ridgefield, CT, United States; Department of Global Computational Biology and Data Sciences, Research and Development Boehringer-Ingelheim, Ridgefield, CT, United States; Department of Gastroenterology and Hepatology, Amsterdam Gastroenterology Endocrinology and Metabolism, Amsterdam UMC, University of Amsterdam, Amsterdam, The Netherlands; Department of Surgery, Amsterdam Gastroenterology, Endocrinology and Metabolism, Amsterdam UMC, University of Amsterdam, Amsterdam, The Netherlands; Department of Gastroenterology and Hepatology, Amsterdam Gastroenterology Endocrinology and Metabolism, Amsterdam UMC, University of Amsterdam, Amsterdam, The Netherlands; Department of Surgery, Amsterdam Gastroenterology, Endocrinology and Metabolism, Amsterdam UMC, University of Amsterdam, Amsterdam, The Netherlands; Department of Gastroenterology and Hepatology, Amsterdam Gastroenterology Endocrinology and Metabolism, Amsterdam UMC, University of Amsterdam, Amsterdam, The Netherlands; Tytgat Institute for Liver and Intestinal Research, Amsterdam Gastroenterology and Metabolism, Amsterdam UMC, University of Amsterdam, Amsterdam, The Netherlands

**Keywords:** perianal fistula, inflammatory bowel disease, wound healing

## Abstract

**Background:**

Perianal fistulas remain a major complication in Crohn’s disease, profoundly impacting quality of life. Although often therapy refractory, strong heterogeneity exists between patients, both in clinical behavior and therapy response. In this study, we aimed to identify biological factors involved in the clinical behavior of perianal fistulas, allowing better stratification.

**Methods:**

Forty-nine patients were included (Crohn’s *n* = 36, cryptoglandular *n* = 13). Gene expression was analyzed in internal fistula openings and tracts by RNA sequencing. Data were verified using spatial transcriptomics and functionally in human adult organoid cultures. A gene-module score for keratinization activity was calculated and correlated to the Crohn’s TOpClass classification as well as to long-term fistula outcomes.

**Results:**

Perianal fistulas were associated with the development of stratified squamous epithelium, expressing specific keratins (KRT5, KRT7, and KRT13). In human organoid models, exposing intestinal mucosa to a pro-inflammatory cytokine mixture partially induced similar changes, supporting rectal mucosal redifferentiation in response to perianal wounding. A gene-module score based on the keratinization-associated markers showed a decreasing gradient from cryptoglandular to mild Crohn-related to severe Crohn-related fistula (0.448 vs. –0.125 vs. –0.448, *P* = .018). Interestingly, the score was also predictive of long-term outcomes: Patients with low keratinization often showed progressive disease (14/20 patients), whereas this was not seen in the high keratinization group (0/16, *P* < .001).

**Conclusion:**

We identify an epithelial redifferentiation process characterized by a keratinization-associated gene module as part of the healing process in perianal fistula. The extent of the keratinization not only distinguished Crohn-related from cryptoglandular fistulas but also aligned with disease severity and healing potential.

## 1. Introduction

Fistula formation is a frequent complication in the course of Crohn’s disease, with up to a third of patients developing 1 or more fistulae during their disease course.^1^ The most common type is perianal fistulas, which profoundly impact quality of life due to fecal loss, abscess formation, pain, and purulent discharge.^2^ In non-inflammatory bowel disease (IBD) patients, cryptoglandular fistulas occasionally occur due to abscess formation in the anal region, mainly in the anal glands. In these patients, treatment through surgical interventions results in high response rates around 80%.^3^ In contrast, Crohn’s disease-related fistulas are highly refractory to therapy, with healing rates usually not exceeding 30%-50%, even using a combination of medical and surgical treatment. Consequently, these patients are at high risk for fecal diversion (ostomy) or even proctectomy further impacting quality of life.^4^^,^^5^

The biology underlying the difference in healing efficacy between Crohn-related and cryptoglandular fistulas remains incompletely understood. Interestingly, neither the occurrence nor the refractory nature of Crohn-related fistulas are correlated to the luminal activity of the disease, suggesting a differential pathogenesis for fistula development and luminal inflammation. Several studies have suggested epithelial to mesenchymal transition (EMT) as a contributor to fistula formation and as culprit in the pathology,^6^^,^^7^ primarily based on the expression of EMT-related proteins including transforming growth factor-beta (TGF-β), SNAIL, and Zinc finger E-box-binding homeobox (ZEB) within fistula tracts. However, expression of EMT related factors was also described in cryptoglandular fistulas, indicating it may be more related to the general wound healing response than have a role specifically in the disturbed healing in Crohn’s disease-related fistulas.^8^

One factor complicating studies into the biology of Crohn-related fistulas is the known heterogeneity within the patient group. Although on average Crohn-related fistulas exhibit reduced healing efficiency, clinical outcomes vary significantly between individuals, resulting in heterogeneous sample sets. To address this, recently a new fistula classification (TOpClass classification) was implemented, which stratifies Crohn’s disease-related fistulas strongly taking into account the likelihood of fistula closure (ie, wound healing).^9^ This stratification enables the formation of more clinically homogeneous subgroups, thereby enhancing the resolution and interpretation of molecular and clinical data. In the present study, we applied the TOpClass classification to account for intra-group variation within Crohn’s-related fistulas and improve the robustness of our analyses.

Here, we show that fistula pathology is marked by morphologic and transcriptional epithelial changes, including altered keratin expression, rather than activation of a full EMT-program. The extent of this process not only distinguishes Crohn-related from cryptoglandular fistulas, but also aligns with disease severity and healing potential. These findings provide a mechanistic explanation for the heterogeneity observed in Crohn’s perianal fistulas and establish a foundation for biomarker-driven stratification and better targeted therapeutic strategies.

## 2. Methods

### Collection of human specimens

Patients suffering from Crohn’s disease related or cryptoglandular fistulas undergoing surgery for their fistula between 2018 and 2023 were included in this study. Crohn’s disease was diagnosed based on clinical, biological, and endoscopic criteria by the treating physician. Crohn-related fistulas were classified according to the TOpClass classification at the time of counseling for fistula surgery at the multidisciplinary outpatient fistula clinic.^9^ Clinical data were collected retrospectively regarding gender, age, diagnosis, IBD-related parameters, and relevant medication (Tables S1-S3, see online supplementary material for a color version of these tables). For follow-up, treatment was provided according to routine care at the physicians discretion. Outcome of treatment was assessed as fistula classification at the latest recorded time point (median follow-up 63 months [range 45-80]).

Samples were obtained during routine care procedures (ie, inspection under anesthesia for Seton placement, surgical fistula closure, or fistulotomy). Curettage material was retrieved from within the fistula tract using a curette surgical instrument to create a fresh tract. The curette is inserted from the external opening and extended into the tract. The sample obtained therefore represents a mixture of the entire length of the tract. The internal opening was identified during surgical proctoscopy (either macroscopically visible or identified by flushing the external opening with saline). Samples were taken from the luminal side, and represent solely the internal opening. Biopsies from normal rectum were obtained from the contralateral rectum equidistal to the anus. For comparative purposes, anodermal resection specimens were collected from separate IBD patients during surgery (proctocolectomy or pouch excision).

For bulk sequencing, samples were snap frozen and stored at −80 °C immediately after collection. Full tissue sections were fixed using 4% paraformaldehyde and subsequently embedded in paraffin blocks.

### Ethics

This study was approved by an Ethics Committee or Institutional Board (Biobank review committee of the Academic Medical Center Amsterdam, [number 178#A201470]). All participants provided written informed consent.

### Bulk RNA isolation and RNASeq

Tissue biopsies and curettage material were homogenized using the SilentCrusher M (Heidolph Instruments) in TRI-reagent (Sigma-Aldrich). Total RNA was extracted using the Bioline Isolate II RNA Mini Kit (Qiagen) according to manufacturer’s protocol. RNA quality was assessed using the Tapestation 4200 (Agilent) and RNA concentration was measured using the Qubit 2.0 Fluorometer (Invitrogen). cDNA libraries were generated using the KAPA RNA HyperPrep Kit with Oligo-dT enrichment (Roche) and sequencing was performed on the Illumina NovaSeq6000. Quality control included Q30 Phred scores, total yield, % of reads mapped to human genome. All QC metrics were comparable between Crohn-related and cryptoglandular fistulas (Figure S1A, see online supplementary material for a color version of this figure).

Publicly available datasets were obtained from the GEO database (GSE117993 and GSE83245, only control subjects from both studies were included). Raw read data was retrieved and processed using the exact same parameters as described above. Comparative analyses were performed using the CLC genomic workbench after normalization and batch correction, efficacy of batch correction is shown in Figure S1B (see online supplementary material for a color version of this figure). For analysis of differentially expressed genes, downweighing of outliers and filter on average expression for false discovery rate (FDR) correction was applied. Pathway analysis was performed by gene set enrichment analysis (GSEA and Fgsea packages in R).^10^

### Immunohistochemistry

Paraffin embedded blocks were sectioned at 4.5 μm, deparaffinized with xylene, rehydrated, and endogenous peroxidases were blocked with 0.01% H_2_O_2_ in phosphate-buffered saline (PBS). Subsequently, slides were boiled in 0.01 M sodium citrate buffer (pH 6) for 10 minutes at 120 °C for antigen retrieval and nonspecific binding sites were blocked using PBS containing 1% bovine serum albumin and 0.1% Triton-X-100 (30 minutes, room temperature). Slides were incubated overnight with primary antibody diluted in the blocking buffer (anti-filaggrin, ab218395, Abcam) and anti-Krt14 (ab7800, Abcam). Brightvision anti-mouseHRP (ready-to-use; Immunologic, VWRKDPVM110HRP) was used as a secondary antibody. Antibody binding was visualized by adding chromagene substrate diaminobenzedine (DAB, Sigma-Aldrich), after which slides were counterstained using hematoxillin (Sigma-Aldrich) and dehydrated and mounted with Entellan (Sigma). Images were obtained using an Olympus BX51 microscope and quantified on a 0-4 intensity scale by a blinded investigator or using Fuji software.^11^

### Digital spatial profiling

Digital spatial profiling (DSP) was performed by GeoMx analysis according to manufacturer’s instructions and as previously described.^12^ In brief, slides with 5 μm FPPE tissue sections were loaded on the Leica Bond RXm system for baking, deparaffinization, and epitope retrieval in ER2 solution (Leica Biosystems, AR9640) for 20 minutes at 100 °C. After incubation for 15 minutes at 37 °C with 1 μg/mL proteinase K (Thermo Fisher Scientific, AM2546) and post-fixation in 10% NBF, the slides were incubated overnight at 37 °C with the human whole transcriptome atlas (WTA) probe panel. Followed by washing the slides in 50% formamide at 37 °C, blocking and staining with antibody for pan-cytokeratin (AE1 + AE3, Novus Biologicals) and nuclear SYTO13 staining (Nanostring, 121303303). After loading and scanning of the slides on the Geomx instrument, the various regions of interest were selected manually based on morphology and a mask for epithelium (pan-cytokeratin positive) was generated to allow capture of the localized probes. Sequencing was performed on a NextSeq sequencer (Illumina).

### Analysis of DSP

DSP data were analyzed using the established GeoMix workflows (GeomxTools, GeoMxWorkflows, and both Nanostring) in R version 4.3.0 and R Studio 2023.03.1. Sequencing reads were converted to expression counts. Preprocessing and QC were performed, using the following parameters: minSegmentReads = 1000, percentTrimmed = 50, percentStitched = 50, percentAligned = 30, percentSaturatio *n* = 50, minNegativeCount = 1, maxNTCCount = 1000, minNuclei = 50, and gene detection rate > 5%. Analysis was limited to genes detected in a minimum of 5% of samples. Data were normalized using Q3 normalization, differential expression was calculated using *t*-test with FDR multiple testing correction. GSEA was performed using GSEA software (version 4.3.2) and gene sets previously described (sets enhanced in fetal vs. adult intestinal epithelium and enhanced in wound associated epithelium vs. normal epithelium).^13^^,^^14^ Prediction of upstream activity was performed using ingenuity pathway analysis (IPA) software (Qiagen). Upstream regulators were restricted to cytokines and growth factors.

For analysis of public data, expression and image data files of perianal Crohn-related fistula were obtained from the study of McGregor et al. (GSE283945).^15^ Data were filtered and normalized using the single cell analysis plugin of the CLC Workbench. Image shown is a specimen with morphologically identifiable intermediate and squamous epithelium (JR7909).

### Organoid cultures

For organoid cultures, colonic biopsies or resection specimens were obtained from 6 adult Crohn’s disease patients (5x non-fistulizing, 1x peri-anal fistula). Samples were washed and the mucosal layer was stripped from the underlying layers. Samples were cut into 5-mm pieces and washed with cold PBS several times until the supernatant was clear. The tissue was incubated in dissociation mix (5 mM EDTA, 2 mM DTT, 1% FCS in Advanced DMEM/F12, 4 °C, 30 minutes). After incubation, pieces were vigorously mixed using a 25 mL pipette, washed in PBS, filtered through a 70 micrometer cell strainer and resuspended in DMEM containing GlutaMAX (Invitrogen), supplemented with penicillin/streptomycin (Gibco) and Hepes (Sigma). Cells were then pelleted, mixed with Matrigel (BD/Corning) and plated in 24-well plates. Isolated colon organoids were cultured in IntestiCult human OGM medium (Stemcell) with 1% penicillin/streptomycin. IntestiCult medium was changed each 3 days and cultured organoids were passaged and expanded every 7 days.

Isolated colon organoids were passaged and allowed to grow out for 3 days. After 3 days, medium was replaced as described below. For differentiation analyses, medium was replaced by IntestiCult medium containing a cytokine mixture (tumor necrosis factor-alpha [TNF-α]) (10 ng/mL, Peprotech, 300-01A), TGF-β (2 ng/mL, Peprotech, cat 100-21) and interleukin-6 (IL-6) (2 ng/mL Peprotech, cat: 200-06). Medium and cytokines were refreshed every 3 days. For 2 donors, images were obtained using an inverted Leica DMi8 Microscope (*n* = 10 images per donor per condition). Image analysis was performed by a blinded observer scoring individual organoids as round/cystic, budded, or flat (examples shown in Figure S2, see online supplementary material for a color version of this figure). Data were calculated as the fraction of each phenotype over all analyzed organoids per image.

### cDNA and quantitative polymerase chain reaction

cDNA was synthesized from 1 μg of purified RNA using RevertAid Reverse Transcriptase according to protocol (Thermo Fisher Scientific, Landsmeer, The Netherlands) in the presence of both Oligo-dT primer and random hexamers primers. Quantitative real time polymerase chain reaction (PCR) was performed using a SensiFAST SYBR No-ROX Kit (Waddinxveen, the Netherlands) according to the manufacturer’s protocol. Primer sequences are listed in Table S4 (see online supplementary material for a color version of this table). Optimal reference genes GAPDH and 36B4 were identified using the GeNorm algorithm and the geometric mean of these was used for normalization of data.^16^ Data are shown as expression relative to the average of control.

### Keratinization module score development

Raw counts were obtained as described above and adjusted for batch effects by negative binomial regression using the ComBat-seq package.^17^ A gene set was selected based on differential expression between Crohn-related and cryptoglandular fistulas, minimal adjusted count of 10 in at least 4 samples, associated with GO pathways involving epidermal differentiation, keratinization, or cornification. This resulted in selection of 18 genes, 15 positively associated (*SPRR2G, PTHLH, SFRP4, KLF7, GATA6, FGF7, LDB2, FGF2, LIPN, LGR4, ROCK1, KRT13, KRT4, KRT1*, and *KRT14*) and 3 negatively associated (*PLAAT4, CALHM6*, and *MYCL*). Adjusted expression counts were normalized for library size and vst transformed using DESeq2.^18^ Gene set scores were calculated using MetaIntegator R.^19^ For dichotomization (high vs. low keratinization), cutoff values were set at the median of all samples.

### Statistical analysis

Statistical analysis was performed using GraphPad Prism v9 or CLC Workbench and the relevant processing packages in R (for RNASeq data). Specific tests per experiment are denoted in the figure legends and included Mann–Whitney *U* test, Kruskal–Wallace with Dunn’s post-test, analysis of variance (ANOVA) with Dunnet’s post-test. Genome-scale testing was corrected by FDR. All tests were performed 2 sided. Data were considered significant with *P* < .05.

### Data accessibility

RNASequencing data are available at 10.5281/zenodo.20557956. Public data are available from GeoDatasets: Bulk RNASeq data, available from GSE117993 (pediatric samples) and GSE83245 (adult samples), spatial data from GSE283945.

## 3. Results

### Patient demographics

Forty-nine patients were included for RNASequencing analysis of the fistula tract curettage (Crohn’s disease *n* = 36, Cryp­toglandular perianal fistulas *n* = 13, Table S1, see online supplementary material for a color version of this table). Median age was 40 years (interquartile range 29-50), and male/female ratio was 26/23. Patients with Crohn-related fistulas were classified according to the TOpClass criteria, and spanned classes 2a-3 (class 2a *n* = 15, class 2b *n* = 8, class 2c *n* = 7, and class 3 *n* = 6). Approximately half the patients had a Seton in situ at the time of sampling (27/49), which did not differ between Crohn-related fistulas and cryptoglandular fistulas.

Within the Crohn’s disease patients, the majority of patients was using IBD-related medication (26/36), in particular biologicals (23/36) and thiopurines (13/36). Details on patient characteristics and medication are summarized in Table S1 (see online supplementary material for a color version of this table).

### Wound healing pathways in CD and cryptoglandular fistulas

**Figure 1.**
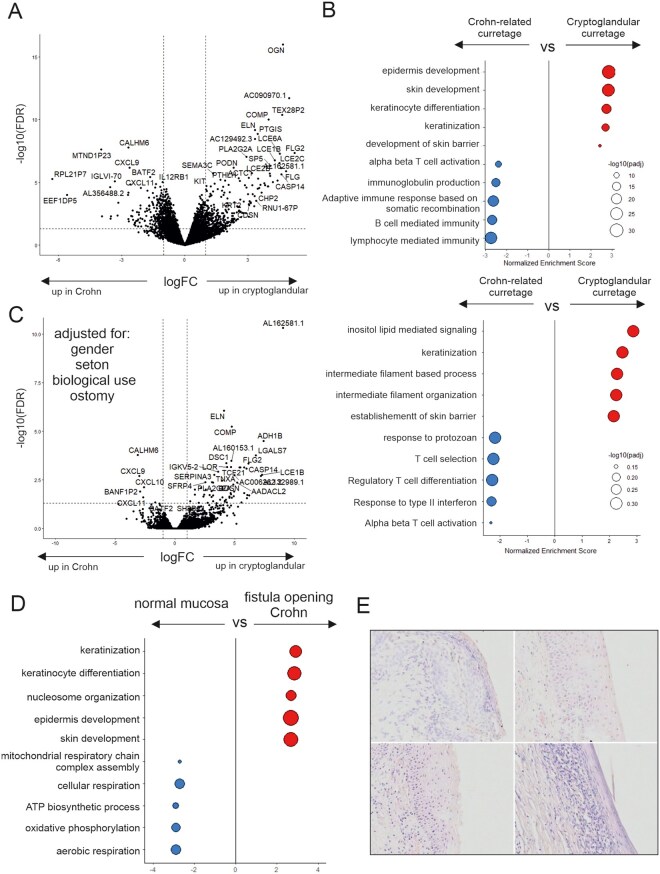
Bulk RNASeq analysis of fistula tracts of perianal fistulas indicates epidermal differentiation as a wound healing response. (A, B) Curettage samples of Crohn’s-related (*n* = 36) and noninflammatory bowel disease/cryptoglandular (*n* = 13) fistulas were obtained and gene expression profiles generated using bulk RNASeq. Pathway analysis was performed using gene set enrichment. (C) Transcriptional and pathway analysis of the same samples as in (A) after correcting for the presence of a Seton, use of biologics, gender, and presence of an ostomy at the time of sampling. Using any (D) pathway analysis comparing biopsies obtained from Crohn-related fistula internal openings and normal rectal mucosa. RNASeq data obtained from noninflamed rectum were obtained from 2 public resources (GSE117993, *n* = 48 and GSE83245, *n* = 21). (E) HE staining of fistula openings from 4 independent patients confirming the presence of stratified epithelium with varying degrees of flattening.

Given the difference in disease course between cryptoglandular and Crohn-related fistulas, we compared fistula tracts (curettage sample) between both groups using RNA sequencing. Overall, 136 differentially expressed genes were identified (FDR < 0.05, Figure 1A). Pathway analysis revealed an increased activation of immune responses, in particular adaptive immune responses, in the Crohn-related fistulas, in line with the underlying immunological condition and with a recent report. In contrast, cryptoglandular fistulas showed increased activity of pathways associated with (epi)dermal differentiation, such as *keratinization* and *epidermis development* (Figure 1B). As treatments vary widely between patients with perianal fistulas, both between cryptoglandular and Crohn-related fistulas and within Crohn-related fistulas, potential confounding factors such as the presence of a Seton, the use of biologicals or the presence of a deviating ostomy as well as gender were assessed. Correction for these factors resulted in comparable results, with increased keratinization/skin development in cryptoglandular fistula and increased immune responses in Crohn-related fistula. However, due to the high levels of correction versus the number of samples, significance was lost in some signals (Figure 1C).

We next assessed whether the keratinization response observed was completely absent in Crohn-related fistulas or just decreased compared to cryptoglandular fistulas. To this end, transcriptional profiles of the internal fistula opening of Crohn-related fistulas were compared to those obtained from normal rectal mucosa, where active wound healing is absent. The most differentially active pathways were again those associated with (epi)dermal differentiation which were now significantly more activated in the Crohn-related fistula openings compared to non-wounded mucosa (Figure 1D), indicating that there is indeed a form of wound healing initiated in the Crohn-related fistulas, albeit less prominent compared to the cryptoglandular fistulas. In line with the transcriptional data, histological assessment of biopsies obtained from the internal opening of the fistula confirmed the presence of stratified epithelium with varying degrees of flattening in the internal openings of fistulas (Figure 1E).

Together, these data imply that keratinization may represent a common repair mechanism in response to fistula formation in both patient groups, with the relative activity of the process differing by underlying diagnosis. Clinically, such gradations could contribute to the observed variation in fistula behavior and refractory nature of Crohn-related perianal fistulas (Figure 1F).

### The wound healing–associated “epidermal differentiation” is distinct from EMT

The very distal location in the rectum raised the question whether the squamous epithelium observed in the perianal fistulas was not simply anoderm and that the transcriptional differences observed were due to a more distal sampling for fistula openings compared to normal rectum. However, immunohistochemistry revealed that although known anoderm-related proteins such as keratin 14 and filaggrin were expressed in the stratified regions in biopsies form the fistula opening, this was much less organized and on a considerably lower level compared to actual anoderm (Figure S3A, see online supplementary material for a color version of this figure). To further exclude the extreme distal location as a potential confounder for the epidermal seen in fistula openings, we compared location-matched samples, that is, a sample obtained from the internal fistula opening and a sample from the contralateral equidistal normal rectum. The internal fistula openings showed increased expression of the keratinization associated genes *SPPR1A* and *CALML5* (Figure S3B, see online supplementary material for a color version of this figure) compared to the normal rectum at the same distance from the anus, further negating a sampling location effect.

**Figure 2.**
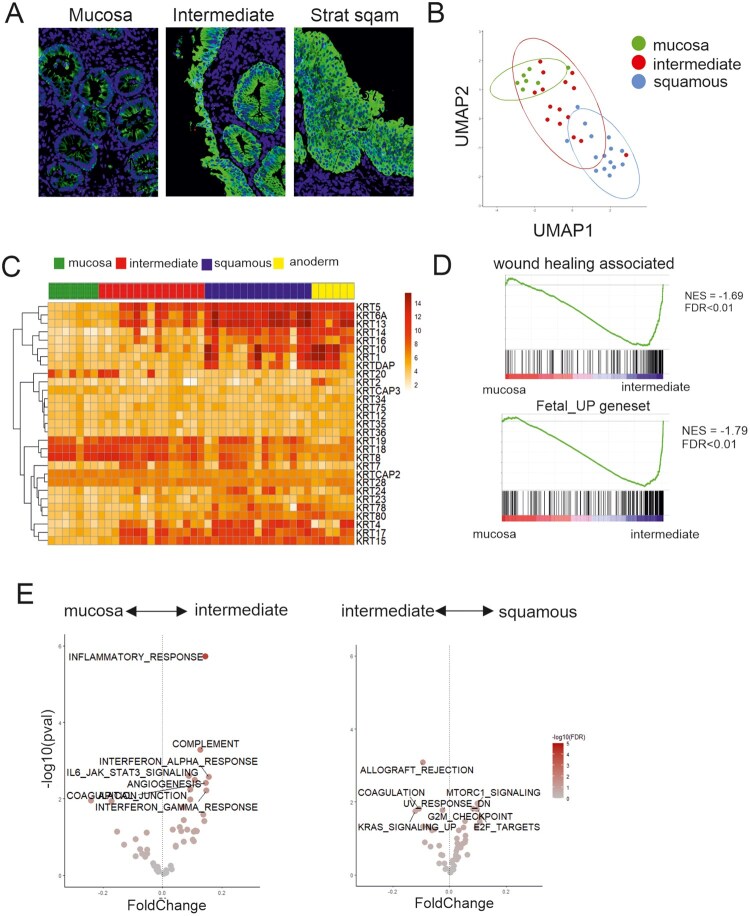
Digital spatial profiling of mucosal, intermediate, and squamous epithelium in fistula openings. (A) Representative image of the 3 types of epithelium selected, stained for pancytokeratin (green) and DAPI (4′,6-diamidino-2-phenylindole, blue). (B) Digital spatial profiling analysis was performed on the mucosal (*n* = 7), intermediate (*n* = 15), and squamous tissue (*n* = 15) in the internal fistula opening using a whole genome library. Clustering by Uniform Manifold Approximation and Projection (UMAP) is shown. (C) Heatmap of expression of keratin transcripts in the 3 subtypes of epithelium. (D) Gene set enrichment analysis of gene sets previously described to be associated with wound healing–associated epithelium (left) or increased in fetal intestine compared to adult (right). normalized enrichment score is shown. (E) Pathway analysis on transcriptional profiles of indicated tissues using the hallmark pathways dataset.

Interestingly, in several samples, the tissue adjacent to the squamous regions was less structured and cuboid, suggesting an intermediate tissue phenotype between columnar and squamous epithelium. To better characterize these epithelial tissue subtypes and establish their mutual relationship, we performed DSP on the fistula openings. Based on morphology, normal appearing columnar mucosa, stratified squamous epithelium, and cuboid intermediate regions were analyzed. Dimensionality reduction of samples showed clear separate clustering of the regions defined as “normal appearing mucosa” and “stratified squamous epithelium” (Figure 2A and B). The cuboid “intermediate” tissue clustered in between the other 2 subsets, supporting not only a morphologic but also transcriptionally intermediate phenotype. For example, the keratin expression profile of the intermediate cells was a combination of the keratins expressed by the intestinal mucosa (eg, *KRT8, KRT18*) and those expressed in the squamous tissue (eg, *KRT5* and *KRT13*). Typical epidermal associated markers such as *KRT1* and *KRT10* were expressed in the squamous areas, however not to the same extend as in the anoderm, which was included for reference purposes (Figure 2C). Recent studies by others have included further spatial analysis of perianal fistula tracts, including epithelium morphologically similar to what is described here.^15^^,^^20^ Overlying the identified keratins on these images suggested a gradual change from epithelium predominantly expressing *KRT8*, to *KRT5*, *KRT13*, and finally *KRTDAP* (Figure S4, see online supplementary material for a color version of this figure).

Previous studies suggested EMT as a key component of fistula formation, based on expression of specific markers. Although some of these markers indeed were expressed in the internal openings (*TGFB1, SNAI2, ITGB6, TWIST1,*  Figure S5A, see online supplementary material for a color version of this figure) as previously described by others, the total EMT program was not specifically activated in any of the tissue subtypes studied here (Figure S5B, see online supplementary material for a color version of this figure).

Re-differentiation of the intestinal mucosa is a form of plasticity which has previously been described in murine models, and was then referred to as “wound associated epithelium” or “fetal-like reprogramming.”^13^^,^^21^ Gene sets associated with these processes were indeed enriched in the intermediate tissue (Figure 2D and Figure S6, see online supplementary material for a color version of this figure). The previous studies suggested a role for the inflammatory response in initiation of this process, which was also supported by pathway analysis in our data, as most pathways differing between normal mucosa and intermediate tissue were related to inflammation (eg, Hallmark pathways “inflammatory response,” “interferon signaling,” and “IL6-Jak-Stat3-signaling,” Figure 2E).

### Human adult mucosal tissue can develop into the intermediate WFDC2+ tissue phenotype

**Figure 3.**
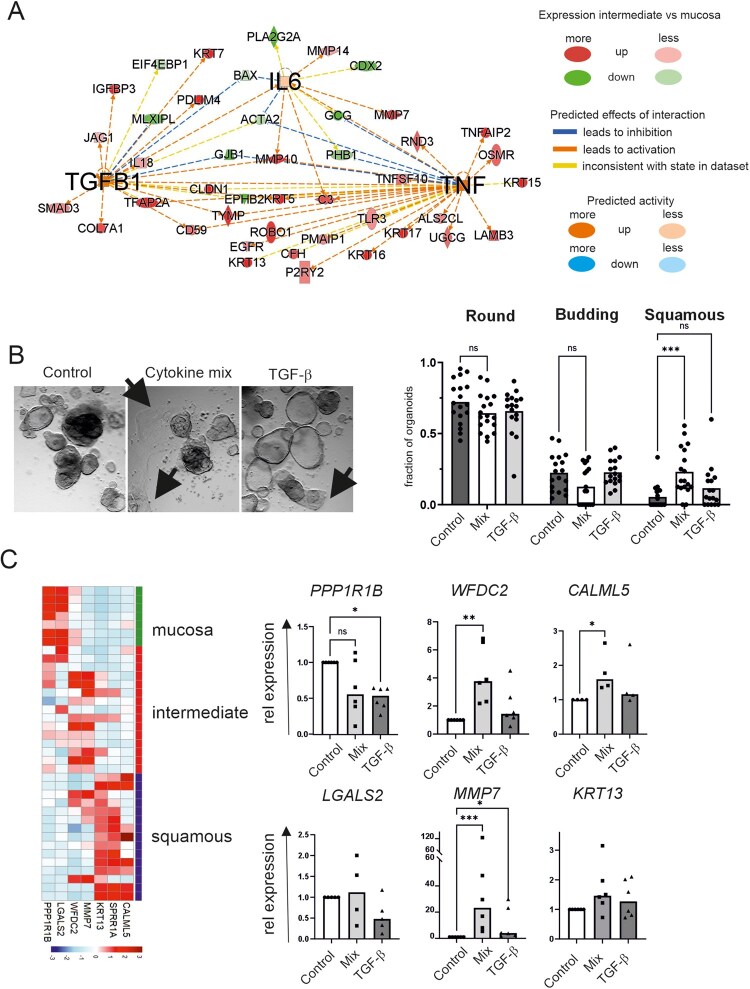
Adult human organoid culture displays transcriptional profile similar to that seen in wound healing. (A) Digital spatial profiling data were used to predict upstream activity in the intermediate tissue type. Expression patterns of intermediate versus mucosal epithelium were compared, and activity of upstream regulators was inferred based on known regulatory profiles and expression patterns of downstream targets. Image depicts the signaling network for tumor necrosis factor-alpha (TNF-α), transforming growth factor-beta (TGF-β) 2 ng/mL, and interleukin-6 (IL-6). (B) Human organoids were obtained from adult donors, and stimulated using a cytokine mixture (TNF-α,10 ng/mL, TGF-β 2 ng/mL, and IL-6 2 ng/mL) or TGF-β alone (2 ng/mL). Subsequently, images were obtained and analyzed for the presence of rounded, budding, or flattened organoids (*n* > 250 cells per condition, 2 individual donors). Bars represent mean, error bars represent SEM, 2-way analysis of variance (ANOVA), corrected for multiple testing by Dunnets. ***P < .001, ns non significant. (C) Left panel displays digital spatial profiling expression profile of the genes selected for organoid culture analysis. Right panel depicts quantitative polymerase chain reaction results shown as relative to the control of the same donor (*n* = 4-6). Bars represent median, dots represent individual experiments of independent donors. Statistics was performed using Kruskall-Wallis test with Dunn’s multiple correction, *P < .05, **P < .01, ***P < .001.

Both the morphology and the transcriptional profile of the intermediate tissue supported a transitional state between the mucosa and the squamous epithelium. To experimentally validate the potential of intestinal epithelium to differentiate into the intermediate and keratinized tissue type observed here, an in vitro model was employed. We performed upstream regulatory analysis using IPA. In this analysis, activity of upstream effectors is inferred based on the differential gene expression patterns observed between mucosal and intermediate epithelium. This analysis suggested among others bioactivity of a network of TNF-α, TGF-β, and IL6. Differential expression of downstream targets of these regulators in the intermediate versus mucosal tissue and predicted interactions in the dataset are shown in Figure 3A. Organoid cultures were established from adult colon mucosa and stimulated using either a mixture of TNF-α, TGF-β and IL6, or TGF-β alone. Stimulation with the cytokine mix resulted in a significantly higher number of organoids with a squamous appearance, in line with the phenotype observed in vivo (Figure 3B). Importantly, TGF-β alone, known to induce EMT, did not have similar effects. Analysis of specific markers that characterized the 3 tissue subtypes in our DSP analyses (Figure 3C, left) showed a strong increase of intermediate transcripts *MMP7* and *WFDC2* and a moderate increase in squamous tissue associated transcripts *CALML5* and *KRT13* although the last failed to reach significance (Figure 3C, right). In summary, the in vitro data show that normal rectal mucosa indeed was able to recapitulate the intermediate and to a limited degree the squamous cell types observed in perianal fistulas upon cytokine stimulation.

### Keratinization levels are related to CD fistula classification and associated to clinical outcomes

**Figure 4.**
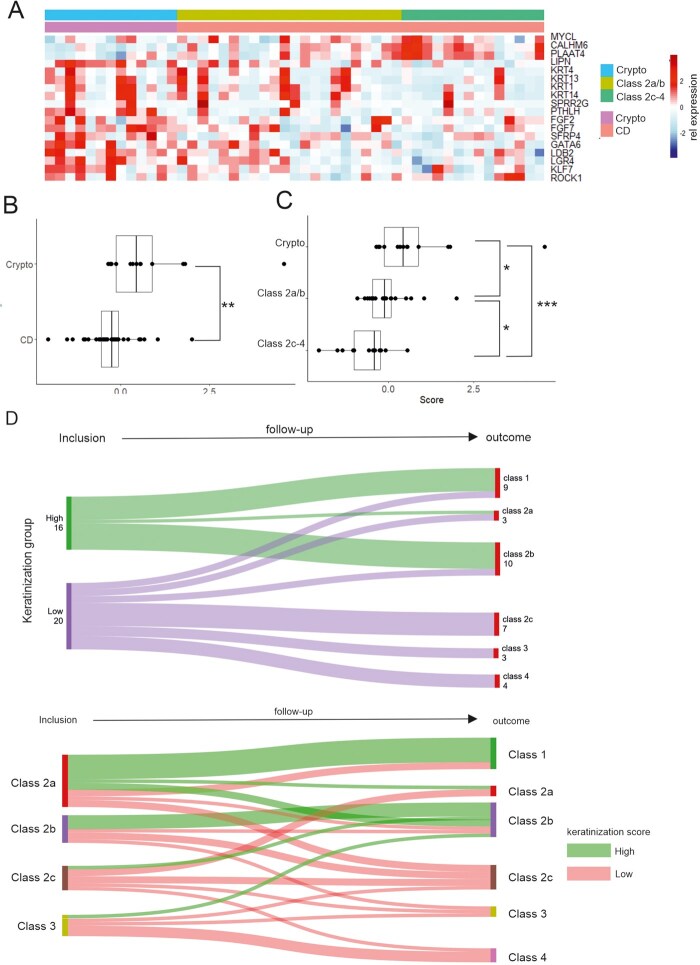
Module scoring of a gene set associated with keratinization associates with diagnosis and outcome of perianal fistulas. (A) Expression of the genes selected for the gene set over individual curettage samples. (B) Gene set scores were calculated based on the RNASeq data of Crohn-related and cryptoglandular fistulas (curettage material). Mann-Whitney *U*-test, **P < .01. (C) Crohn-related fistulas were classified as (rapid) progressive (TOpClass class 2c or higher) or milder, Kruskall-Wallis test with BH correction, *P < .05, ***P < .001. (D) Calculated scores associated to the outcome of the fistula using current standard of care with a median follow-up of 63 months. (E) TOpClass classification at time of inclusion separated into keratinization score high and low associated to the outcome of the fistula using current standard of care with a median follow up of 63 months.

Based on the observation that keratinization was more pronounced in cryptoglandular than in Crohn-related fistulas, we sought to determine whether the degree of this process could be quantified and linked to differentiation between the 2 different fistulas and to clinical behavior. Based on the earlier differential expression data obtained in the curettage material (Figure 1), a subset of genes related to epidermal development was selected. Visualization shows expression of these genes indeed differs between cryptoglandular and Crohn-related fistulas (Figure 4A). This was quantified by calculating a compound weighted “keratinization module” score for this gene set for each individual sample. Cryptoglandular fistulas clearly showed higher scores compared to Crohn’s-related fistulas (Figure 4B, median 0.448 vs. −0.321, *P* = .0018). Recognizing that Crohn’s-related fistulas are clinically heterogeneous, with the recently proposed TOpClass classification system differentiating (rapid) progressive fistulas (class 2c and higher) from relatively more mild phenotypic disease, we compared keratinization scores across these subgroups. Keratinization was significantly higher in class 2a/b cases compared to class 2c-4 (Figure 4C, –0.125 vs. –0.448, *P* < .02).

Interestingly, the fistula keratinization score was strongly correlated to the outcome of the fistula upon receiving current standard of care. After a median follow-up of 63 months (range 45-80), patients with a low keratinization score at inclusion developed (rapid) progressive disease requiring further interventions (class 2c-4) significantly more often than patients with a high keratinization score (14/20 [70%] vs. 0/16 [0%], *P* < .001, Figure 4D). In particular, patients with a low score at inclusion numerically received more proctectomies during follow-up (4/20 vs. 0/16). Of note, even when subdividing patients based on TOpClass classification at the time of inclusion, within each class those with a high keratinization score tended to have better outcomes than those with low scores (Figure 4E).

## 4. Discussion

In this study, we show that perianal fistulas involve the development of squamous epithelium expressing specific keratins (eg, *KRT5, KRT13*). In Crohn’s disease patients, the potential to develop this epithelium is heterogeneous, and directly correlates to the TOpClass fistula classification and the subsequently healing perspective. These findings provide a biological basis for the clinical observation that some fistulas appear more epithelialized and are more amendable to surgical closure, whereas others remain refractory.

In earlier studies, EMT has been suggested as a major driver of fistula formation, but in this study, we did not detect a clear activation of the EMT program.^6^^,^^7^ Although this may appear contradictory, it is also a matter of interpretation. Much of the previous work focused on a limited number of EMT-associated markers, and showed these were upregulated in fistulas when compared to normal mucosa. Indeed, when focusing on individual markers such as decreased *CDH1* (E-Cadherin), or increased *TGFB1* and *ECM1*, we did corroborate these earlier findings. However, expression of just a few genes associated with EMT does not constitute the occurrence of the phenomenon of EMT as a whole, which is also supported by the EMT International Association in their guidelines.^22^ When evaluating the full arsenal of EMT-associated genes, no clear activation could be seen in any of the tissue types in fistulas, suggesting the previous studies may have highlighted more of a general wound response (which includes many of the same molecules as EMT) rather than full EMT.

A recent study challenges the previously presumed role of TGF-β in fistula formation, as the authors show decreased TGF-β responsive signatures in the rectal mucosa of Crohn’s disease patients with perianal disease compared to those without.^23^ In their study, biopsies were obtained from explicitly non-inflamed rectal mucosa rather than involved areas. This design allowed the authors to assess changes in the rectal mucosa of patients with perianal disease in the absence of inflammation, and thus identify signals present prior to fistula initiation. In our study, we evaluated samples obtained from the fistula itself (either internal opening or curettage material), providing data on biological activity once wounding has occurred. Combining these datasets would generate a model where prior to damage, the rectal mucosa already shows alterations creating a more damage-permissive state, for example by increased epithelial TL1A expression and subsequent alterations in immune and stromal cells. This state is not mediated by enhanced TGF-β signaling, but may even reflect relative resistance to this cytokine. Upon damage of the rectal mucosa, the physiological response would be to initiate a wound healing program, including epithelial redifferentiation under influence of TGF-β and other cytokines. Although this process was observed in both Crohn-related and cryptoglandular fistula, it can be easily envisaged that the relative TGF-β resistance observed in Crohn’s disease patients results in a less effective redifferentiation.

The in vitro model presented here indicates the capacity of the intestinal mucosa to transition into phenotypes resembling the intermediate and squamous epithelium seen in fistula in vivo. However, as this was a proof-of-principle study, we did not assess the full transcriptome in order to pinpoint the exact overlap. Interestingly, the decrease in mucosal-associated transcripts was most pronounced by TGF-β stimulation alone, while the increased expression of intermediate and squamous markers was significantly stronger upon stimulation using the cytokine mixture. This may indicate that the loss of mucosal markers mainly depends on TGF-β, while the induction of intermediate and squamous transcripts requires additional inflammatory cytokine signaling. Moreover, additional combinations of cytokines or alterations in timing and dosing may result in a more complete capture of the transitioning process, which is a topic of current investigation. In this study, the dual comparison of Crohn-related fistulas to both normal (non-wounded) mucosa and cryptoglandular (relatively well-healing) fistula tracts, identified the development of the squamous stratified tissue as a component of wound healing. This is in line with clinical observations by gastrointestinal surgeons who frequently describe cryptoglandular fistulas amenable for surgical closure as “more sturdy.” Despite this standing knowledge, until now no biological basis was available for this phenomenon, impeding development of objectifiable biomarkers or even interventions in the process. The data presented here provides a gene set allowing better stratification of perianal fistulas. This stratification is relevant, as studies have shown early intervention in perianal fistulas is associated with significantly better patient outcomes.^24^ In contrast, due to the high disease burden of non-responsive perianal fistulas, early recognition of fistulas with little to no chance of closure may warrant more advanced interventions at an earlier stage without the current delay. Finally, a relevant proportion of patients presents with perianal fistulas as the first symptom of Crohn’s disease. This group is likely to be treated rather conservatively as cryptoglandular, increasing the delay between symptom onset and effective intervention. A recent manuscript identified red flags based on clinical characteristics.^25^ The addition of biomarkers such as described here may further improve early diagnosis of fistula first Crohn’s disease, although further studies will be required to evaluate sensitivity.

In addition, better understanding of the biological pathways impacted in poorly healing perianal fistulas may open new avenues for therapeutic intervention. Given the inflammatory component in Crohn-related fistulas, described by others as well as here, anti-inflammatory therapies have understandably been at the forefront, in particular those also used in luminal inflammatory bowel disease. However, based on the unique tissue characteristics of wound healing in the rectal area, active stimulation of wound healing mechanisms may be an additional treatment target. As an example of this, hyperbaric oxygen therapy has recently gained interest in the therapy of refractory perianal fistulas, with promising results.^26^ Preliminary data from our group show that hyperbaric oxygen therapy shifts Crohn-related fistula patients with a refractory phenotype to a state more amenable to surgical fistula closure. Interestingly, this was associated to an increased activation of the same keratinization/epidermal differentiation pathways described here.^27^

In summary, the wound healing response in perianal fistulas includes development of squamous epithelium expressing specific keratins which is partly impaired in Crohn’s disease-related fistulas. These data provide a basis for better stratification of perianal fistulas requiring early intervention, as well as generate leads for new therapeutic interventions.

## Supplementary Material

jjag092_Supplementary_Data
