# Supplementary material for: Impaired keratinization activity in perianal Crohn’s fistulas is associated with poor prognosis
Source: J Crohns Colitis. 2026 Jun 30;20(6):jjag092. doi: 10.1093/ecco-jcc/jjag092 (PMC13318234; doi:10.1093/ecco-jcc/jjag092)
Supplement: jjag092_Supplementary_Data [file jjag092_supplementary_data.docx]

**Supplementary material**

**Supplementary figures S1-S6**

**Supplementary tables S1-S3**


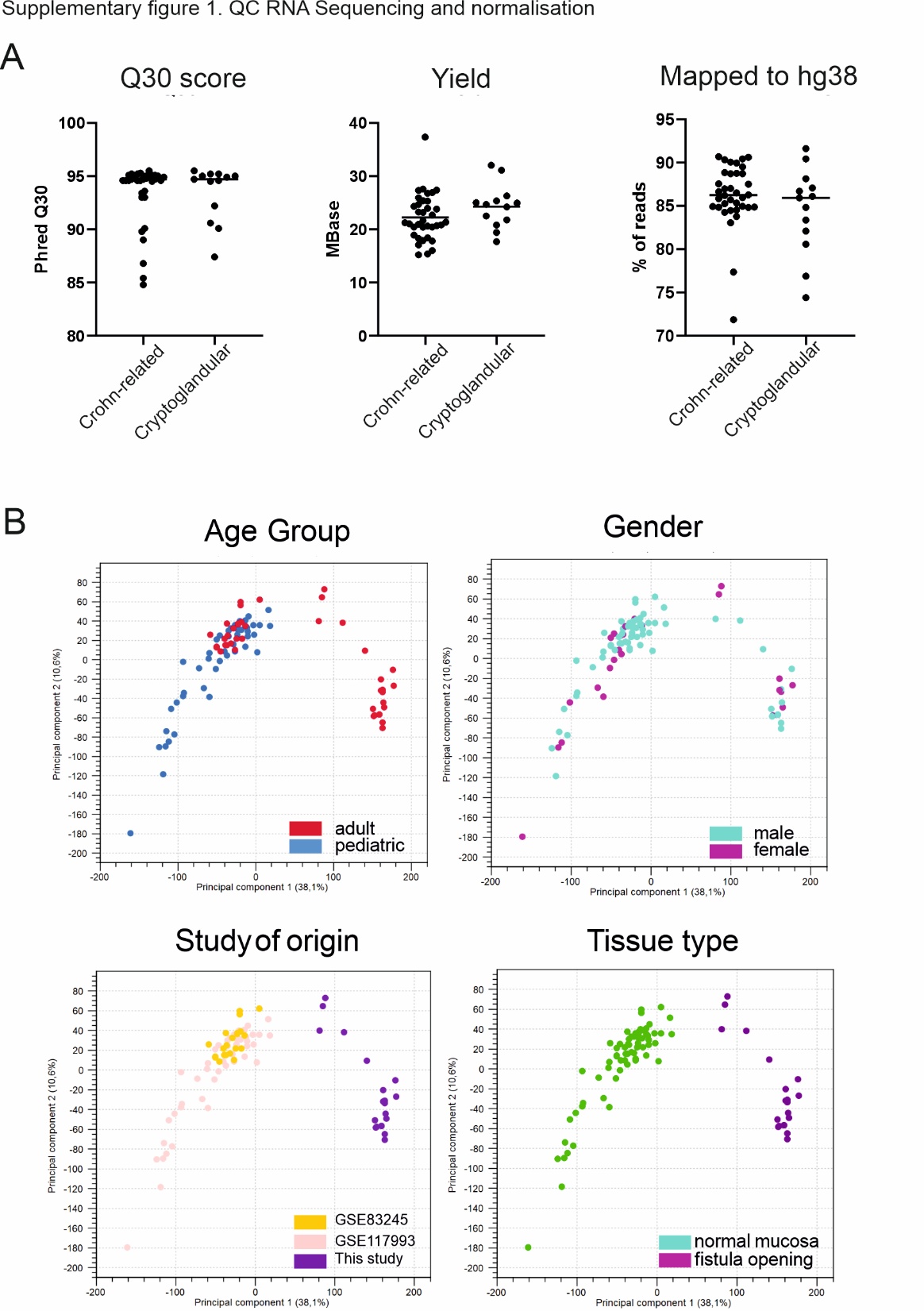


**Figure S1.** (A) Quality parameters of RNASeq. QC metrics of the RNASeq procedure (library prep, sequencing and alignment for individual RNA samples. (B) PCA plots depicting the clustering of samples obtained from two independent public datasets (rectal biopsies) as well as this study (biopsies of internal fistula opening). Primary separation was due to the type of tissue used (normal mucosa vs fistula opening)rather than age group (pediatric vs adult), gender or the experiment of origin of the data, indicating effective batch correction.


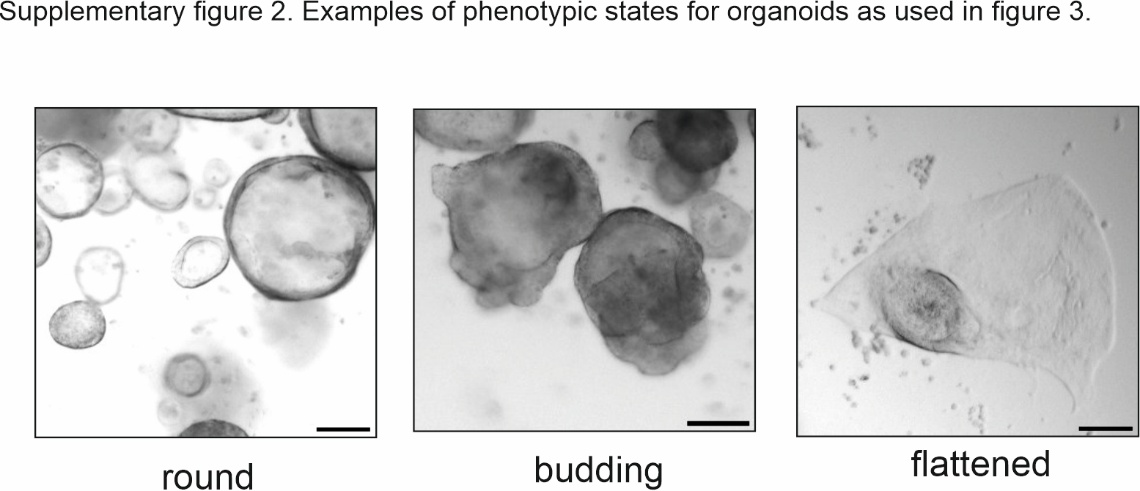


**Figure S2. Examples of organoid scoring as used in Figure 3.** Images of organoid cultures were scored by a blinded observer, where each organoid was assigned as either round (left), budding (middle) or flattened (right) based on appearance as shown here. Scale bar depicts 100 micrometer.


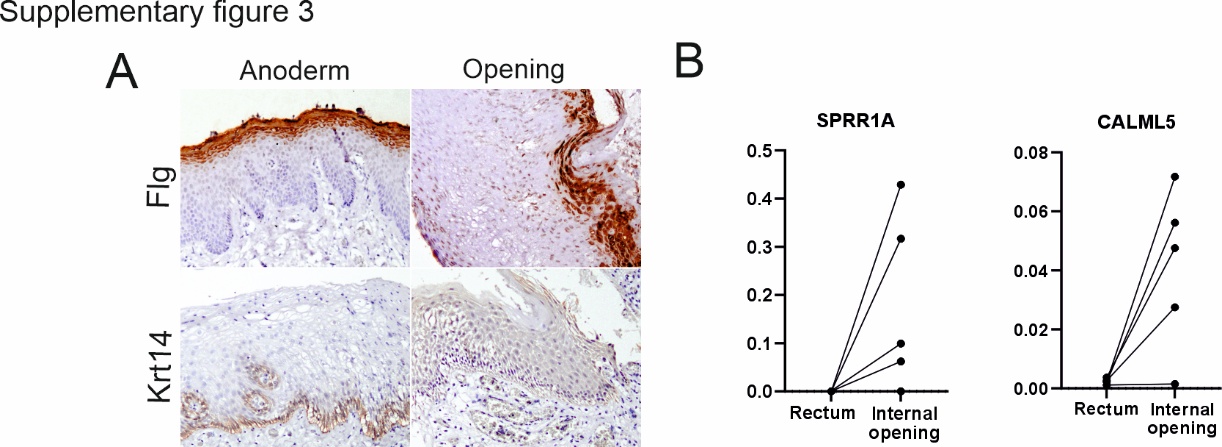


**Figure S3. Stratified squamous tissue in fistula openings is not fully congruent to anoderm.** (A) Biopsies of anoderm (n=2) and internal openings (n= 7) were obtained from IBD patients and stained for Fillagrin (Flg) and Keratin14 (Krt14). Representative image shown. (B). Matched biopsies were obtained from the internal fistula openings and contralateral rectum at equal distance from the anus. Expression of squamous tissue associated transcripts was determined by rt-pcr.

**
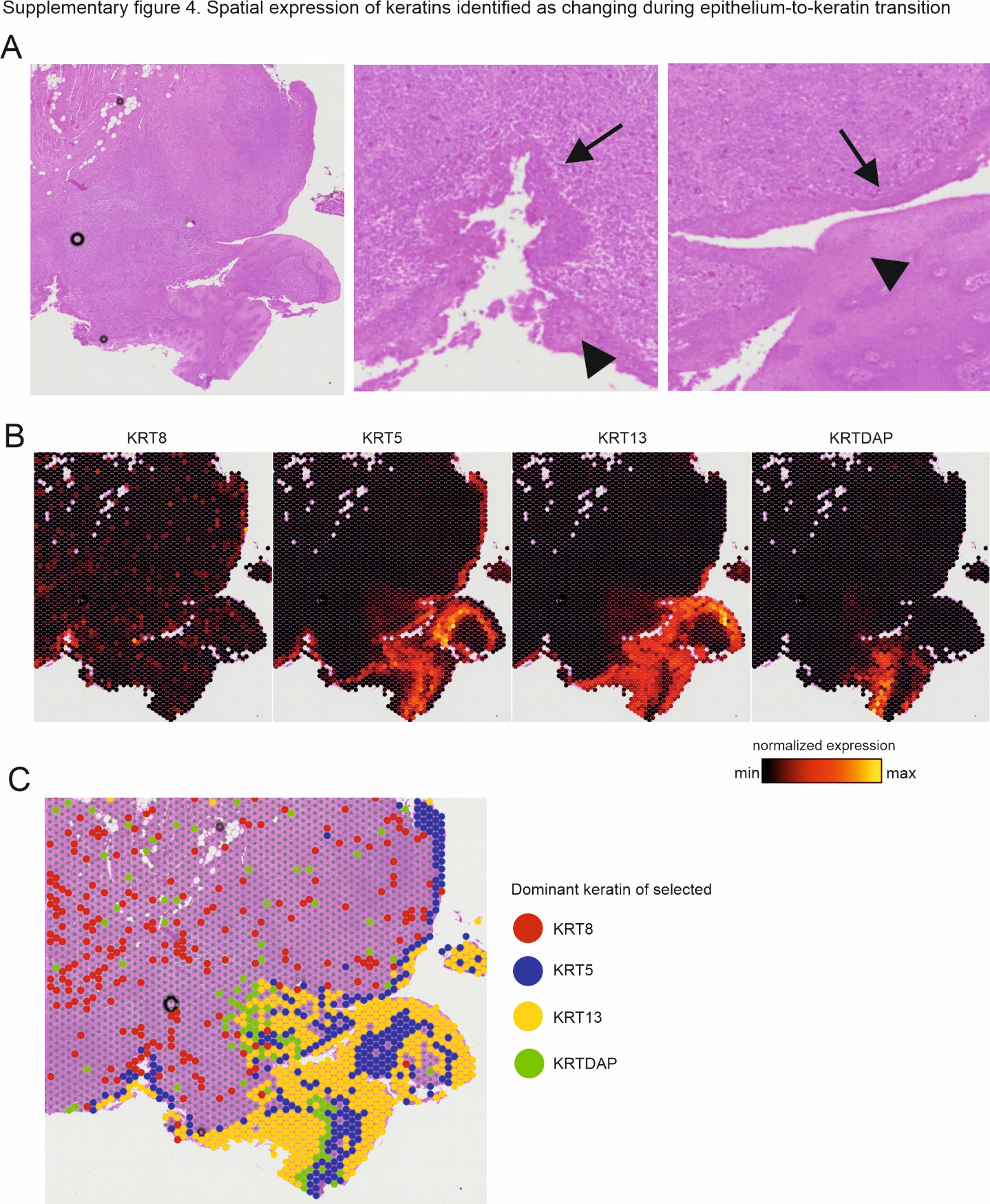
**

**Figure S4. Expression of keratins identified as changing during epithelium-to-keratin transition.** (A) Data was obtained from the study published by McGregor et al, and a tissue section of a perianal fistula containing morphologically intermediate (arrows) and squamous epithelium (arrowheads) was identified. (B) Expression of selected keratins which were shown to be altered between mucosa, intermediate and squamous epithelium in our DSP analysis is depicted.(C) The same tissue section depicting the predominant keratin of the four shown in panel B over tissue locations. A gradual change from *KRT8* to *KRT5, KRT13* and finally *KRTDAP* can be observed.


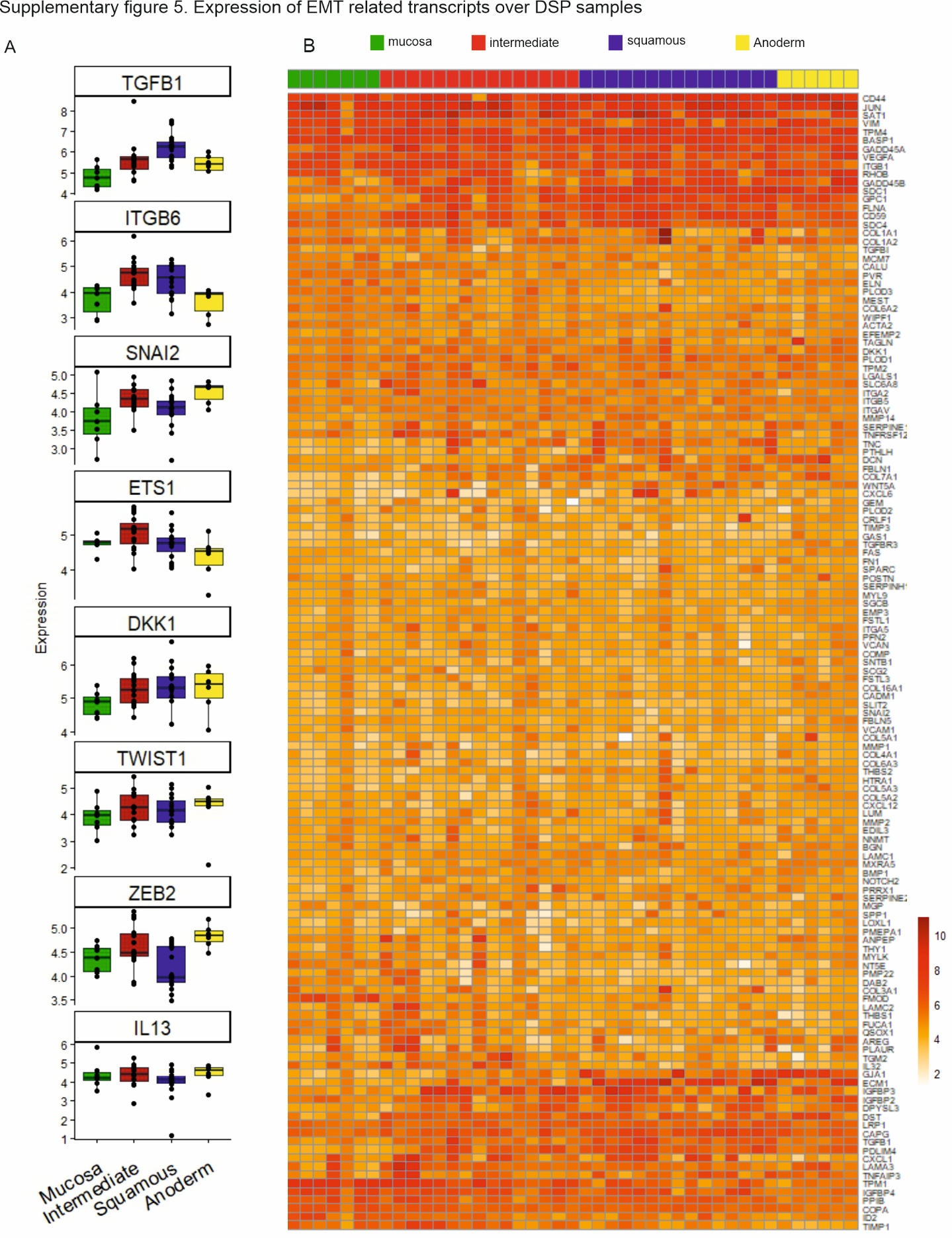


**Figure S5. Expression of EMT related transcripts over DSP samples.** DSP analysis was performed on biopsies of the internal fistula openings as shown in main Figure 2. Epithelium was identified by pancytokeratin staining, and based on morphology mucosal (n=7), intermediate (n=15) and squamous tissue (n=15) regions were selected. Separate samples were included for anodermal tissue (n=6). Analysis was performed (n=6) using a whole genome library. (A) Expression patterns over epithelial tissue subtypes of EMT markers previously associated with fistula formation. (B) Expression is shown for the genes included in the Hallmark geneset ‘*Epithelial to Mesenchymal Transition’* and passing quality control in the DSP analysis. Genes were clustered unsupervised.


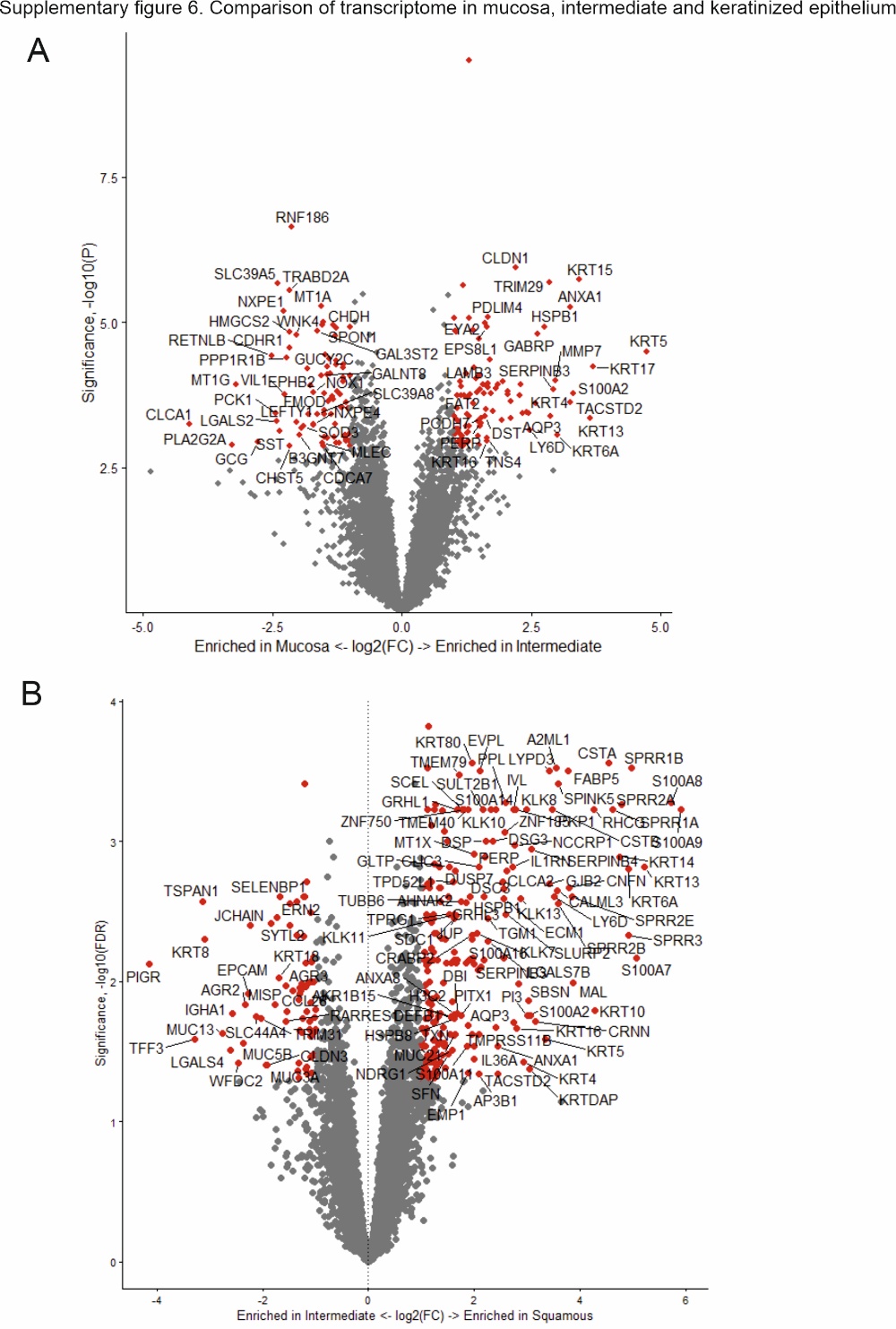


**Figure S6. Comparison of transcriptome in mucosa, intermediate and keratinized epithelium.** Digital spatial profiling was performed internal fistula openings. Regions were designated ‘mucosa’, ‘intermediate’ and ‘keratinized’ based on positive staining for PanCK and morphology. Comparative analysis of mucosa (n=7) vs intermediate (n=15) and intermediate (n=15) vs keratinized (n=15) regions is shown as volcano plots. Red dots depict FDR<0.05.

Supplemental Table 1. Baseline characteristics of patients included in RNA-Seq analysis of fistula tracts

Supplemental Table 2. Baseline characteristics of patients included in analysis of fistula openings

Supplemental Table 3. Baseline characteristics of patients included in spatial analysis

Supplementary Table 4. Pcr primer sequences
